# Remarks on Bandaging the Abdomen after Delivery

**Published:** 1852-09

**Authors:** J. H. Beech

**Affiliations:** Coldwater, Mich


ART VII.— *Remarks on Bandaging the Abdomen after Delivery.* By J. H. Beech, M. D.

I was surprised to see an article in the *Buffalo Med. Journal,* "On Bandaging the Abdomen after Delivery," without editorial comment in reference to the sentiment. We believe the article calculated to mislead students and inexperienced practitioners, in regard to the importance of the bandage in question, and that serious consequences might result to many while experiments were establishing facts which are almost universally received by the profession.*

Either Mr. Kesteven or myself have certainly misunderstood the object expected to be gained by the bandage, and we believe that physicians generally have no such idea of the "rationale of its usefulness" as he supposes. We think few expect to "promote the contraction of the uterus by the bandage," but rather to maintain the contraction, by supplying the accustomed pressure of the abdominal walls, until the muscles shall have regained their power of contracting upon the viscera alone. We find bandages necessary for the comfort and safety of patients, whenever we abstract fluids from the thoracic cavity, from hydrocele or anasarca, after evacuating large abscesses, and whenever we remove fluids or solid substances from the abdominal cavity of males or females.

We have never ventured to apply a bandage to a puerperal woman, nor leave her until the uterus was firmly contracted; but after this, the bandage properly applied, reaching from the ensiform cartilage to the os pubis, laced or pinned smoothly, is usually a comfortable means of supplying the place of that tension which had been so suddenly lost by parturition, and without which faintness and exhaustion are apt to be experienced.

Without this support, we believe the uterus is inclined to become relaxed, the fatigue of its contractile tissue allowing it to yield to the lochial

---

* We do not limit our selections of eclectic matter to articles in which the views expressed accord with our own. We concur fully with Dr. Beech in his reasons for the use of the bandage.—*Editor Buff. Med. Jour.*

exhalation, until the veins of the placental surface became patulous, and blood begins to ooze, or flow *in uteri*, in proportion to the dilatation.

If the uterus makes efforts to arrest the flow by regaining its contracted condition, the pains (after pains) will be more or less severe in proportion to the neuralgic condition of the uterus, and to the firmness or size of the coagulum to be expelled.

It is no uncommon circumstance to see patients express great relief from syncope and distress upon the proper adjustment of a bandage, even where the uterus had kept firmly contracted.

The muscles of the "*laboring poor*" contract more readily than those more easily circumstanced, and they are not so apt to suffer from neglecting the bandage; but in this country they are far from being ignorant of its use and value. We understand that even the Indians formerly inhabiting this vicinity made use of such support.

We have had no experience with "pads, basins, &c., &c.," except so far as to have seen enough to satisfy us, that they may be so used as to interfere with the very object intended; but removing such, have found, after proper preliminary treatment, that a suitable bandage placed the patient in a better condition to leave in the care of a nurse.

I am very respectfully yours, &c.,

COLDWATER, MICH., *July* 15, 1852. J. H. BEECH, M.D.

